# Three-Dimensional Histology Volume Reconstruction of Axonal Tract Tracing Data: Exploring Topographical Organization in Subcortical Projections from Rat Barrel Cortex

**DOI:** 10.1371/journal.pone.0137571

**Published:** 2015-09-23

**Authors:** Izabela M. Zakiewicz, Piotr Majka, Daniel K. Wójcik, Jan G. Bjaalie, Trygve B. Leergaard

**Affiliations:** 1 Department of Molecular Medicine, Institute of Basic Medical Sciences, University of Oslo, Oslo, Norway; 2 Department of Neurophysiology, Nencki Institute of Experimental Biology, Warsaw, Poland; University of Pennsylvania Perelman School of Medicine, UNITED STATES

## Abstract

Topographical organization is a hallmark of the mammalian brain, and the spatial organization of axonal connections in different brain regions provides a structural framework accommodating specific patterns of neural activity. The presence, amount, and spatial distribution of axonal connections are typically studied in tract tracing experiments in which axons or neurons are labeled and examined in histological sections. Three-dimensional (3-D) reconstruction techniques are used to achieve more complete visualization and improved understanding of complex topographical relationships. 3-D reconstruction approaches based on manually or semi-automatically recorded spatial points representing axonal labeling have been successfully applied for investigation of smaller brain regions, but are not practically feasible for whole-brain analysis of multiple regions. We here reconstruct serial histological images from four whole brains (originally acquired for conventional microscopic analysis) into volumetric images that are spatially registered to a 3-D atlas template. The aims were firstly to evaluate the quality of the 3-D reconstructions and the usefulness of the approach, and secondly to investigate axonal projection patterns and topographical organization in rat corticostriatal and corticothalamic pathways. We demonstrate that even with the limitations of the original routine histological material, the 3-D reconstructed volumetric images allow efficient visualization of tracer injection sites and axonal labeling, facilitating detection of spatial distributions and across-case comparisons. Our results further show that clusters of S1 corticostriatal and corticothalamic projections are distributed within narrow, elongated or spherical subspaces extending across the entire striatum / thalamus. We conclude that histology volume reconstructions facilitate mapping of spatial distribution patterns and topographical organization. The reconstructed image volumes are shared via the Rodent Brain Workbench (www.rbwb.org).

## Introduction

Deciphering the complex wiring patterns that underlie brain function and behavior has been an important topic of research for over more than a century [1, 2]. Axonal tracing studies have contributed importantly to the mapping of neuronal projections, by identifying presence of connections between different regions of the brain, elucidating strength of projections, demonstrating shape and size of terminal fields of axons from tracer injected sites, and mapping topographical organization [3–7]. Precise mapping of the wiring patterns of the brain and identification of the organizing principles underlying the spatial distribution of neural connections is furthermore relevant for ongoing large scale efforts to better understand brain function through computational modeling [8, 9].

Classical tracing studies involve tissue processing and analysis of histological sections sampled from regions of interest [10]. By collecting series of sections and performing a systematic analysis, several important features related to projection patterns can be revealed. An additional level of precision and a more complete visualization and improved understanding of complex topographical relationships is achieved with use of three-dimensional (3-D) reconstructions [11–15]. For smaller regions of the brain, 3-D reconstruction techniques have been combined with use of local coordinate systems [16] for integrating data from a large number of tract tracing experiments [17]. Aggregation of large numbers of spatially registered connectivity data in a local coordinate system, serving as a standardized spatial framework, was instrumental for revealing wiring patterns and topographical principles of organization in cerebro-ponto-cerebellar networks [7, 14, 18–20]. In these studies, aspects of the observed labeling patterns were recorded as spatial points patterns [21], followed by visualization and analyses. While this approach was manageable for smaller brain regions, reconstructing the entire brain in 3-D with manual or semi-automatic recording of labeling patterns would not be practically feasible.

With the recent introduction of robotic microscope systems and slide scanners, high-resolution images of histological sections can be accumulated and shared via online database systems [22, 23]. Public collections of 2-D tract tracing image data [24] are well suited for mapping of the location of axonal connections across the entire brain [25], while further detailed analyses of projection patterns and topographical organization require 3-D reconstruction. Reconstructing high resolution images of histological sections into volumetric images provides several advantages, such as virtual slicing of the volumetric representation in different angles [26–28].

In this study, we explore to which extent 3-D reconstructions of a comprehensive collection of publicly available high resolution images [24], acquired originally only for microscopic analysis of single sections, can be used for investigating projection patterns and topographical organization across multiple brain regions. We present volumetric reconstructions of histological tract tracing data showing somatosensory projections in the rat brain. Axonal tracers were placed in whisker and forelimb representations of the primary somatosensory cortex (S1) and the series of section images demonstrating the ensuing labeling were reconstructed in 3-D. We demonstrate how 3-D reconstructed histological images facilitate visualization and analysis of projection patterns and topographical organization, and provide examples of observations from the volumetric reconstructions that are not readily seen in the series of 2-D sections. Examples include comparisons of the spatial location and extent of tracer injection sites, and the size, shape, and spatial organization of labeled corticostriatal and corticothalamic axonal clusters.

## Materials and Methods

Serial image data from four tract tracing experiments available in the Whole Brain Connectivity Atlas application (http://www.rbwb.org/) were used. Detailed experimental procedures are given in the original data publication [24]. Briefly, an anterograde axonal tracer (biotinylated dextran amine, BDA), was injected in forelimb and whisker representations of S1 in anaesthetized adult Sprague Dawley or Wistar rats. After 7 days animals were sacrificed and transcardially perfused with 4% paraformaldehyde, and brains were removed for histological processing. 50 μm thick coronal sections were cut on a freezing microtome. Every second section was processed to visualize BDA [29] and counterstained with Neutral red. High-resolution section images (TIFF format) were obtained through a 10× objective (Olympus UPlanApo, NA 0.40) using a motorized Olympus BX52 microscope running the Virtual Slide module of Neurolucida 7.0 (MBF Bioscience Inc., Williston, VT, USA). Images were converted to the Zoomify PFF format (Zoomify Inc., Santa Cruz, CA, USA) and assembled in an online data repository. The location of tracer injection sites were confirmed by analysis of anatomical landmarks and cytochrome oxidase staining pattern [24]. In the online viewer application, images are spatially organized according to serial order with stereotaxic coordinates assigned.

**Fig 1:**
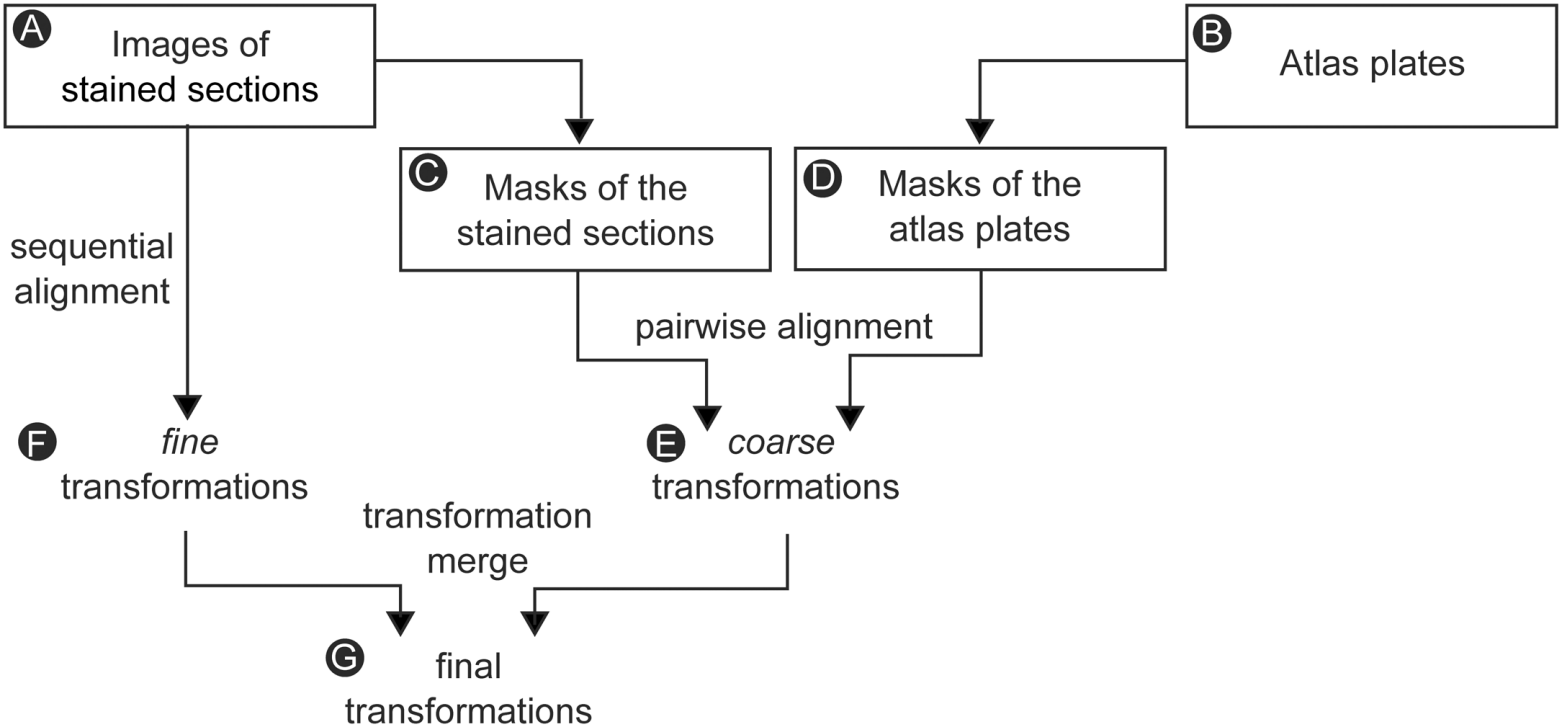
3-D reconstruction workflow. Flowchart showing the processing steps for 3-D reconstruction of histological images. See text for details.

Complete series of images of BDA and neutral red sections (spaced at 100–200 μm) from four cases were downloaded in bulk, and 3-D reconstructed using a customized workflow (Fig 1). To streamline computations, images were downsampled to 15 μm per pixel. For each section image (Fig 1A), the best corresponding atlas plate (Fig 1B) was identified on basis of anatomical landmarks. Image masks (Fig 1C) were semi-automatically generated by smoothing the red channel of each slice image with a 5 × 5 pixel median filter, with a threshold at 95% of the maximum image intensity, before applying minor manual corrections to refine the masks and remove spatially inconsistent or displaced tissue elements. The spatially consistent regions were typically the striatum, brainstem, thalamus, superior colliculus and pons, while the cerebral cortex, parts of the hippocampus and cerebellum were often displaced during histological processing and excluded from the mask. The section image masks were used to create correspondingly masked atlas plates (Fig 1D) by use of the ITK-SNAP software application [30].

For each case, the series of sections images were reconstructed into volumetric form following a two-step approach [30] with *coarse* and *fine* rigid transformation steps using the Advanced Normalization Tools (ANTs) software package [31]. Co-registration of image volumes was achieved using a common volumetric reference space that was reconstructed from a standard stereotaxic rat brain atlas [32] by use of the 3D Brain Atlas Reconstructor digital atlasing tool [33] (http://3dbar.org, 3dBAR). The initial *coarse* registration relied on aligning section masks (Fig 1C) to correspondingly masked atlas plates (Fig 1D). It was performed by minimizing sum of squared differences and yielded series of *coarse* rigid transformations for each block (Fig 1E). The subsequent *fine* alignment step was performed using section images. Sections were sequentially aligned using rigid transformations, starting from the middle section of each block for each specimen towards either end, using cross correlation as similarity metric. The series of *fine* transformations (Fig 1F) were subsequently convolved with a Gaussian kernel (with σr = 20 sections for the rotation angle and σt = 5 for translation parameters) to extract the low frequency component which was then filtered out. The procedure was repeated for each block from each specimen before the obtained sets of combined final transformations (Fig 1G) were applied to the original section images. Following 2-D alignment of section images to corresponding atlas plates, each image was assigned axial position values according to the calculated distance from bregma and mapped using linear interpolation. The assembled image volumes were viewed using ITK-SNAP, allowing orthogonal views of the 3-D reconstructed labeling. Image brightness and contrast were adjusted to enhance visibility of axonal tracers using the Brightness/Contrast command in Adobe Photoshop CS5 (Adobe Systems Inc. San Jose, CA, USA).

Geometric surface models of tracer injection sites and labeled axonal plexuses in the striatum and thalamus were made by manual segmentation of filtered binary images in which pixels representing BDA labeled fibers were extracted from background staining. Native red, green, blue (RGB) color mode images were adjusted for unequal brightness, contrast, and saturation using standard filters available in Adobe Photoshop. RGB channels providing the most contrast between signal (BDA labeling, green channel) and background (Neutral red staining, red channel) were selected for further processing. The red (background) channel was selected as a mask and subtracted from the green (signal) channel. The section images of each individual specimen were reconstructed into volumetric form using transformations computed during the previous step and then mapped into the atlas reference space.

The volumetric image data generated in this study are available for inspection and downloading via the Rodent Brain Workbench (www.rbwb.org).

## Results

We have 3-D reconstructed publicly available series of histological images sampled from four rat brains in which focal injections of the axonal tracer BDA were placed in the whisker (n = 2) and forelimb (n = 2) representations of S1 (www.rbwb.org; [24]). Our aim was to explore the added value of reconstructing volumetric image data for investigations of topographical distribution patterns in brain networks. Further, the image volumes were combined in a common spatial reference space, and axonal labeling in two major target regions for S1 projections (striatum and thalamus) was delineated and surface rendered for evaluation and comparison of spatial distribution patterns. We here exemplify how these image data can be utilized to characterize and compare the 3-D shape and spatial organization of tracer injection sites, and labeled axonal clusters in major target regions for S1 projections.

### Spatial distribution of tracer injection sites

**Fig 2:**
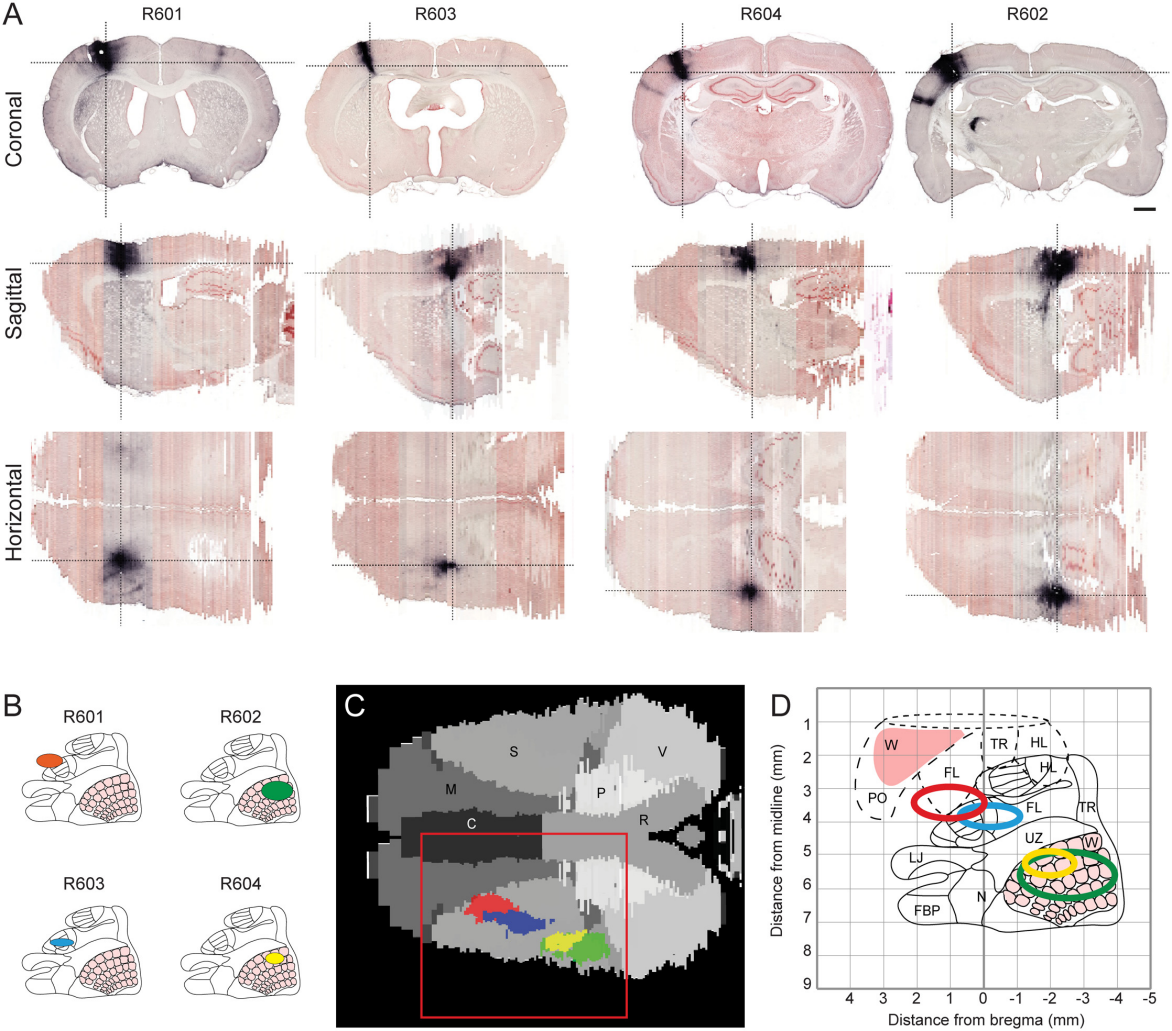
3-D reconstructed injections combined in atlas template. Analysis of the spatial location of tracer injection sites in the four cases investigated. (A) Coronal, sagittal, and horizontal slices through the injection site centers at the level of cortical layer V, facilitating comparison of the spatial location and extent of the four tracer injections. Dashed lines indicate slice locations in corresponding images. (B) Cartoon representations of the primary somatosensory cortex (redrawn and modified from [34] with permission) showing the estimated locations of the tracer injection sites. (C) Horizontal slice through the 3-D reconstructed atlas (at level of layer V) showing the relative location and extent of the co-registered delineations of the four injection sites. (D) Estimated stereotaxic positions of injection sites projected onto a composite map of M1 and S1 based on several earlier electrophysiological studies (redrawn and modified from [59] with permission). This map indicates that the stereotaxic location of the tracer injection in case R601 to a larger degree involves the forelimb representation in M1, rather than S1. C, cingulate cortex; FBP, furry buccal pad; FL, forelimb; HL, hindlimb; LJ, lower jaw; M, motor cortex; N, nose; P, parietal cortex; PO, perioral; R, retrosplenical cortex; S, somatosensory cortex; TR, trunk; UZ, unresponsive zone; V, visual cortex; W, whisker; Scale bar, 1 mm.

The value of axonal tract tracing data critically depends on the characterization of the location and spatial extent of tracer injection sites. As described in the original publication presenting the material [24], these tracer injections were targeted to whisker and forelimb representations in S1 (Fig 2B), to cover all layers of the cerebral cortex, and involve both cytochrome oxidase positive barrels and adjacent septa [25]. The spatial location was validated by inspection of anatomical landmarks and evaluation of cytochrome oxidase positive barrels in S1. The spatial coordinates of injection site centers were assigned using a standard rat brain reference atlas [32]. While the size and extent of injection sites are readily observed in single section images, the spatial relationship among injection sites is more difficult to evaluate. We therefore utilized the volumetric image data to visualize the four injection sites in three orthogonal planes (Fig 2A) in relation to the reference atlas space (Fig 2C). These images provide an overview of the spatial location, extent, and relationship among the four cases investigated. A horizontal slice through the volumetric atlas space showed that the injection sites were primarily distributed along the anteroposterior axis, with substantial overlap between the two injections in the S1 barrel cortex, and little overlap between the two injections placed in the S1 forelimb representation (Fig 2B and 2C). When further evaluating the spatial distribution of axonal labeling, we noticed that the labeling originating from the most anteriorly located forelimb-related tracer injection (R601) was differently distributed compared to the other S1 forelimb case (R603), as detailed below. The spatial location of the injection site center (1 mm anterior and 3.5 mm lateral of bregma), is indicated as part of the S1 forelimb representation in the originally employed reference atlas [32], but according to more detailed functional maps of the S1 cortex based on electrophysiological recordings [34], this location more likely involves the forelimb representation of the adjacent primary motor cortex (M1, Fig 2D). Thus, the tracer injection in case R601 most likely involves the forelimb representations in S1 and the adjacent M1. This may explain the deviating topographical distribution of labeling in this case, and otherwise serves to illustrate the importance of carefully considering the spatial distribution of tracer injection sites when interpreting axonal tracing data, and perhaps most importantly that certain anatomical positions can be assigned different identity in different reference resources.

### Observing axonal labeling in volumetric histology images

The volumetric images from the four reconstructed and co-registered cases were inspected in spatially synchronized three-plane ITK-SNAP viewers allowing efficient evaluation and comparison of the spatial distribution of labeling. A major advantage of the volumetric imaging is dynamical browsing, allowing visual tracking of the labeling through multiple image volumes. In this way, the segregated trajectories of whisker and forelimb related projections through the external capsule, internal capsule and cerebral peduncle are easily observed (not shown). The same dynamic three-plane viewing technique also facilitated the assessment of the 3-D shape of the labeled axonal clusters. High density labeling was observed in the volumetric images whereas individual fibers, low labeling densities, and cytoarchitectural features were better observed in the individual high-resolution section images. The results were influenced by the spatial registration accuracy. Thus, labeling patterns were readily observed in multiplane images from brain regions with the highest registration accuracy (forebrain and midbrain) and less clearly demonstrated in regions with lower registration accuracy (hindbrain and brain stem). For the present analysis of topographical organization, we focused on regions with relatively high registration accuracy, in particular the abundant projections to the striatum and the thalamus [25]. We also observed labeling patterns in the pontine nuclei that were in agreement with earlier descriptions [14, 18, 25], with axonal clusters in spherical, lamellar subspaces [35]. However, the spatial resolution and accuracy of registration was not sufficient to allow a useful study of topographical organization in this part of the brain.

### 3-D shape and spatial distribution of labeled corticostriatal and corticothalamic projections

**Fig 3:**
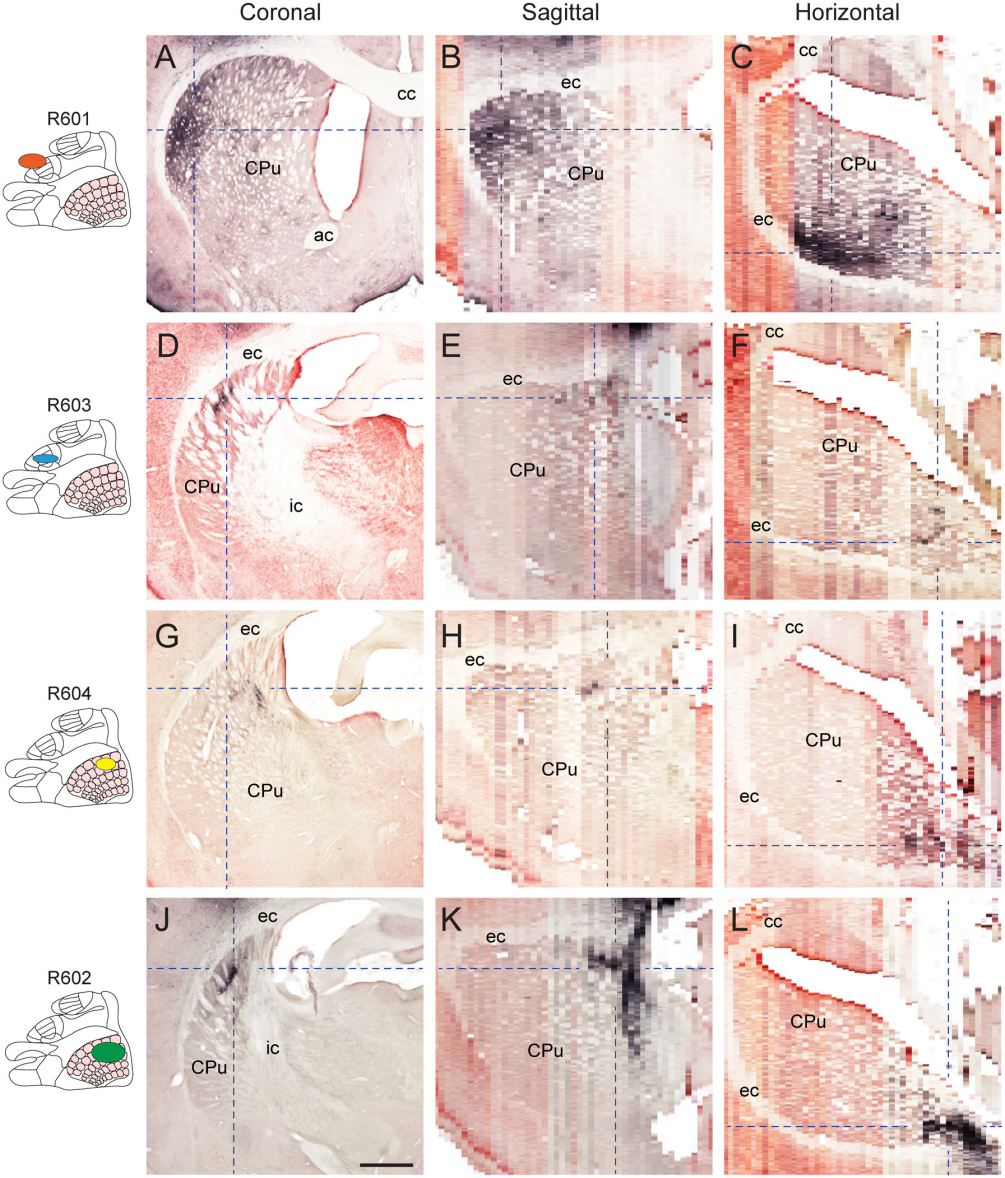
3-D shape and distribution of corticostriatal projections. Visualization of axonal labeling in three-plane slices centered on clusters with highest labeling density in the striatum of the four cases investigated. Insets (left) indicate the location of tracer injections in S1 (presentation as in Fig 2). Dashed lines indicate slice locations in corresponding images. ac, anterior commissure; cc, corpus callosum; ec, external capsule; ic, internal capsule; Cpu, caudate-putamen complex (striatum). Scale bar, 0.5 mm.

Axonal projections from the S1 barrel cortex to the striatum are known to terminate in one or more elongated, slightly curved axonal clusters in the dorsal striatum [36, 37]. These characteristics were also observed in our reconstructed images, with relatively small, narrow plexuses located in the dorsal part of the posterior striatum (Fig 3). The most prominent corticostriatal labeling was observed in case R601 in which the injection site involved forelimb representations in M1 and S1. In this case, labeled axons were distributed in an extensive and dense plexus of labeled axons, distributed in a wide, curved, lamellar subspace (Fig 3A–3C). Notably, this labeling was located in more anterior and lateral parts of the striatum, relative to the projections originating from the other injection sites in S1.

**Fig 4:**
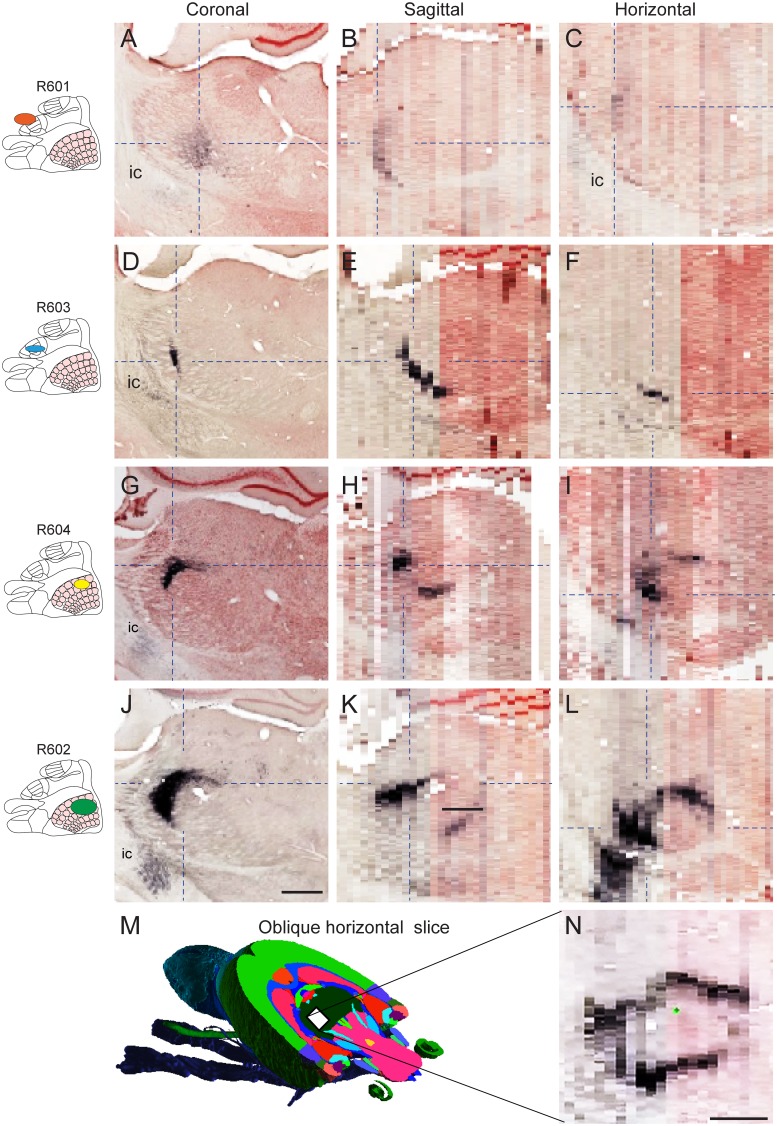
3-D shape and distribution of corticothalamic projections. Visualization of axonal labeling in three-plane slices centered on clusters with highest labeling density in the thalamus of the four cases investigated (A-L). Insets (left) indicate the location of tracer injections in the primary somatosensory cortex (presentation as in Fig 2). Dashed lines indicate slice locations in corresponding images. ic, internal capsule. (M) Oblique horizontal slice through a volumetric rat brain atlas [57] indicating the orientation of an arbitrary slice through an image volume (N), showing an almost continuous ring of axonal labeling in the thalamus (case R602). Scale bars, 0.5 mm.

Corticothalamic projections from S1 barrel cortex are known to terminate in sharply defined clusters of laminar or cylindrical shape, somatotopically distributed in thalamic subregions [38–43]. In the coronal images available in the online atlas (Fig 4A, 4D, 4G and 4J), corticothalamic fibers were found in several sharply defined clusters distributed across the reticular thalamic nucleus, ventral posterolateral and posteromedial nuclei, posterior thalamic nuclear group, and the ventral anterior and ventrolateral thalamic nucleus. Contrary to the labeling observed in the striatum (Fig 3), the highest densities of thalamic labeling was seen in the three cases injected in S1 (Fig 3D–3L), while the labeling in case R601 (M1/S1, forelimb) was more restricted, more medially located, and of lower density compared to the other cases (Fig 4A–4C). When this labeling pattern was dynamically inspected in three image planes (Fig 4), labeled clusters were found to be distributed within a spherical layer. This pattern was particularly apparent for projections originating in the S1 barrel cortex (Fig 4G–4L). Moreover, when slicing the volumetric images in non-standard planes (Fig 4M), the axonal labeling was seen to form a near continuous ring-like shape within the thalamus (Fig 4N). This pattern suggests that corticothalamic fibers are distributed according to a common organizing principle across the different thalamic subregions.

### Topographical organization in the striatum and thalamus

**Fig 5:**
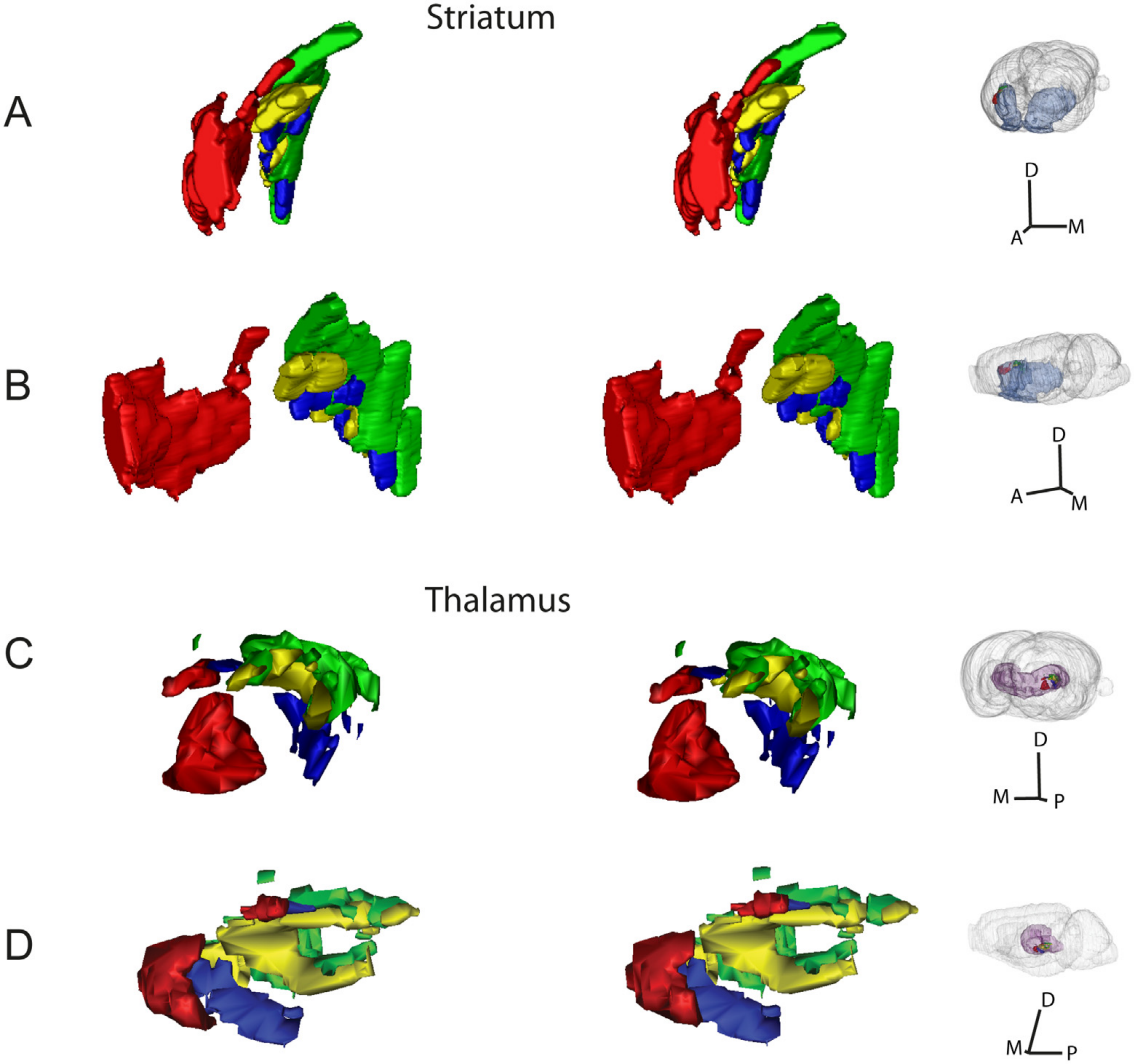
3-D surface models of corticostriatal and corticothalamic projections. 3-D visualization of topographical organization in corticostriatal and corticothalamic pathways. (A-D) Stereo-image pairs showing color coded 3-D surface models of the delineated axonal clusters in the striatum (A,B) and thalamus (C,D), in slightly offset views from anterior (A), medial (B,D), and posterior (C), as indicated in the inset figures (right column). To perceive the 3-D images the viewer must cross the eye axis to let the image pairs merge. (A,B) Clusters representing corticostriatal axons are distributed within a narrow, laminar subspace, extending in an anteroposterior direction through the dorsolateral striatum. (C,D) Clusters representing corticothalamic axons are distributed within a narrow, spherical shell-like subspace in the thalamus. A, anterior; D, dorsal; M, medial; P, posterior.

We have prepared geometric surface models demonstrating the shape and size of the labeling in each experiment. Since all data were registered to the same atlas space, differences in distribution could be readily observed by dynamic viewing or use of stereo image pairs (Fig 5).

In the striatum, the three injections placed in S1 gave rise to elongated and flattened clusters that were partly overlapping and confined to a narrow, lamellar subspace, and slightly shifted along the anteroposterior axis (Fig 5A and 5B). The 3-D renderings (Fig 5A) nicely reproduce the pattern of interdigitating clusters described in an earlier tracing study using dual tracer injections [44]. By contrast, the labeling originating from the M1/S1 forelimb representation (R601) was distributed in a large and separate lamella-shaped cluster more anteriorly located in the striatum. Inspection of the combined data in 3-D showed that all clusters were distributed in a narrow lamellar subspace extending through the dorsolateral striatum from anterior to posterior. In the thalamus, the multiple, widespread clusters of labeling attained curved, lamellar shapes of different size. Dynamic rotation of the 3-D reconstructions showed that most of the clusters are distributed within a large, spherical subspace, resembling an onion layer (Fig 5C and 5D). The projections from the two overlapping injections in the S1 barrel were also overlapping in the thalamus (Fig 5C and 5D; yellow and green clusters), but with the projections from the largest and most posterolaterally located injection (R602, green clusters in Fig 5) slightly shifted towards external. The projection originating in the S1 forelimb representation (Fig 5, blue clusters), was distributed in segregated, more anteriorly and ventrally located clusters, while the injection in the M1/S1 forelimb representation (R601) gave rise to clusters of labeling that were more medially and anteriorly located, in the anterior “cap” of the onion-like subspace (Fig 5, red clusters). Anteromedially, an additional group of clusters are found in a more external subspace.

The findings thus demonstrate that clusters of corticostriatal and corticothalamic projections are systematically distributed within confined lamellar subspaces spanning across the entire striatum and thalamus, and exemplify how 3-D reconstructed volumetric histology images facilitate investigations of topographical organization.

## Discussion

We have reconstructed 3-D image volumes from standard serial section images and explored the value of histology volume reconstructions by investigating spatial distribution patterns and topographical organization of S1 corticostriatal and corticothalamic projections identified in the reconstructed image volumes. Compared to investigations in single 2-D images of this type of material, our results show that the 3-D reconstructions 1) improve the evaluation of tracer injection sites considerably, 2) are well suited for investigation of strong axonal labeling and detection of differences in spatial distribution, and 3) are less suitable for investigations of single fibers, weak labeling, and cytoarchitectonic boundaries. Analyses of topographical organization is facilitated by superimposing data from multiple experiments in the same coordinate system and performing 3-D surface modeling of the distribution of labeling.

The histological material employed for this study consisted of a series of section images with a spacing of 100–200 μm between sections. The material was originally generated for the purpose of 2-D atlasing and not for 3-D reconstruction, and may as such be considered to be representative for a standard series of section images, in line with data generated in most laboratories working with axonal tract tracing techniques. Our results showed that adequate image registration was achieved in most parts of the brain even without use of external reference images, as evident from the distinctly coherent labeling that could be traced through the digitally created sagittal and horizontal image slices. In spatially inconsistent brain regions, i.e. ‘peripheral’ regions that were more distorted during histological processing, the linear registration methods were less successful. By masking out such regions from the reconstruction, registration improved in regions of primary interest located centrally in the forebrain. We further attempted to use more advanced non-linear registration methods [27, 45](see, also [28, 46]), but this primarily improved visual appearance in certain regions, while introducing inappropriate anatomical distortions in regions with curved geometries (results not reported). This lack of success can likely be ascribed to well-known challenges related to the relatively large spacing between consecutive sections in our material [47]. Higher registration accuracy might have been achieved by registration of sections to block-face images acquired during histological sectioning [28] or to an MRI template [48]. Nevertheless, within the limitations inherent to current methodology and the material employed, our approach worked well for large parts of the brain. Our results thus allowed us to demonstrate how 3-D reconstructions of serial sections generated with routine sectioning procedures yield considerable added value by allowing efficient image browsing and examination of complex distribution patterns in multiple planes. Careful evaluation of tracer injection locations is important in all analyses of tract tracing experiments.

The present results demonstrate that registration of images to a common atlas template provides helpful visualization of the relative position of tracer injection sites, which is important for interpretation of topographical distribution patterns. While topographical displacements of injection sites and labeling patterns are readily identified in serial 2-D histological images, 3-D reconstructions facilitate detection of patterns. A further point of interest is that functional interpretations of spatial locations vary across reference atlases or maps, such that the same spatial location (i.e. the stereotaxic coordinates of center of the tracer injection in case R601 used in our study) is interpreted as part of S1 in one atlas [32], and as part of M1 in a different map [34]. While this point was of little importance for our previous brain-wide mapping of presence of labeling in this material [25], it was more relevant when other features of labeling, such as spatial distribution and topographical organization as evaluated in the present study. Given that reference maps will inevitably be adjusted or refined over time in response to new knowledge, it follows that assignment of well-defined 3-D spatial coordinates to observations is of importance for data mining and re-interpretation of public data collections.

Subcortical projections are characterized by complex spatial distribution patterns. Spatial principles of organization are therefore difficult to discern by inspection of 2-D image series. The use of 3-D reconstructions that can be interactively inspected from various perspectives, or image volumes that can be digitally sliced in arbitrary angles, are instrumental for detecting complex distribution patterns, such as the lamellar and circular patterns seen in the present study. Similar conclusions were reached in earlier studies of complex clustered distributions of corticopontine projections in monkey [49], cat [50], and rat [14]. Our analyses of corticostriatal and corticothalamic projections from the S1 barrel cortex indicate that subcortical projections from the sensorimotor cortex are distributed in widespread clusters that are systematically organized in lamellar subspaces. Similar principles of organization have been demonstrated in the pontine nuclei, and have there been interpreted to reflect maturational gradients [7]. In addition, the volumetric images revealed salient differences in the spatial distribution and density of S1 forelimb and whisker related projections to the striatum and thalamus. Our 3-D reconstructions thus indicate that forelimb and whisker related projections are more closely associated in the striatum than in the thalamus, reflecting the lattice organization described for sensory corticostriatal projections [44]. By contrast, projections from M1 are located in distinctly different parts of a common (imaginary) lamellar subspace. Viewed in multiple planes, the volumetric images also showed to advantage the distinct differences in axonal density, where the density of S1 projections was considerably higher in the thalamus compared to the striatum, while this was opposite for M1 projections. These differences likely reflect different signal processing and different contributions of the striatum and thalamus to motor and sensory related functions. It should, however, be noted that the large scale distribution patterns described here, include projections from cortical barrels as well as adjacent septa, and thus overarch finer levels of compartmental organization described in the striatum [51, 52] and thalamus [41, 42].

Novel methods for acquisition of tract tracing data in 3-D image volumes at cellular resolution are becoming increasingly available. A recent example is serial two-photon tomography [53], a new method instrumental for collecting the large collection of 3-D tract tracing data available in the Allen Mouse Brain Connectivity Atlas [54]. This unique resource of volumetric data from more than 2000 tract tracing experiments accumulated in a common atlas space is a major breakthrough for the mapping of connections in the mouse brain. But, although such image acquisition methods and large public repositories will undoubtedly facilitate future connectivity mapping efforts in the mouse brain, similar resources are not yet available for the rat, beyond the limited atlas provided via the Rodent Brain Workbench [24]. Further, since few laboratories have the resources required to conduct such large-scale investigations, we believe that conventional tract tracing approaches will remain relevant. In this respect, procedures for 3-D reconstruction and registration of histological image to common atlas space represent a way to make conventional collections of tract tracing data available to the community. Efforts in this direction are undertaken by large infrastructure projects, including the Human Brain Project, aiming at integration of data generated by many laboratories in a common framework [55, 56]. Through such efforts, collections of existing histological tract tracing data, representing valuable resources for the community, could be brought together and made available for re-analysis in a common volumetric atlas space.

A further advantage of 3-D reconstructed histology volumes is that comparison with the new volumetric tract tracing data repositories is facilitated. Thus, when homologous tracer injection locations are looked up in the Allen Mouse Brain Connectivity Atlas (http://connectivity.brain-map.org/), the patterns of labeled corticostriatal, corticothalamic, and corticopontine projections seen there are highly similar to those observed in our study. If this atlas is queried for experiments conducted in wild type C57/Bl6 mice with tracer injections placed in the barrel field or upper limb representations of S1, six experiments are found. Interactive inspection of the volumetric images from these selected cases in three-plane image online viewers, reveals large, elongated bands of labeling in the striatum, and dense, curved clusters of labeling in the thalamus, together forming ring-like patterns, similar to those observed in our 3-D reconstructions in rat. Such patterns are particularly notable in experiments with large tracer injections (experiments 112951804; 126909424 in the Allen Mouse Brain Connectivity Atlas). Thus, spatial distribution patterns found by investigating the volumetric data from the Allen Mouse Brain Connectivity Atlas are remarkably similar to those observed in our volumetric material from the rat.

We conclude that 3-D reconstructions of histological image data bring considerable advantages to the mapping of spatial distributions and topographical organization, while detection of labeled fibers to determine presence of connections requires use of original high-resolution images [25]. Ongoing work will expectedly provide practical procedures for registration of various histological section data to open access volumetric atlas templates recently made available for the rat brain [57, 58]. We envision that future data repositories will consist of numerous, spatially registered tract tracing data. With tools for online browsing and selective co-display of different cases (to some extent available in the current version of the Allen Institute Mouse Brain Connectivity atlas), it will be possible to more efficiently map principles of topographical organization simultaneously across multiple brain systems.
